# Correction: No interaction between fundamental-frequency differences and spectral region when perceiving speech in a speech background

**DOI:** 10.1371/journal.pone.0250918

**Published:** 2021-04-27

**Authors:** The PLOS ONE Staff

## Notice of republication

An incorrect version of the Abstract and Introduction was published in error. This article was republished on April 16, 2021, to correct for this error. The publisher apologizes for the error. Please download the article again to view the correct version. The originally published, uncorrected article and the republished, corrected article are provided here for reference.

## Supporting information

S1 File Originally published, uncorrected article. (PDF)

S2 File Republished corrected article. (PDF)
