# Supplementary material for: Correction: No interaction between fundamental-frequency differences and spectral region when perceiving speech in a speech background
Source: PLoS One. 2021 Apr 27;16(4):e0250918. doi: 10.1371/journal.pone.0250918 (PMC8078814; doi:10.1371/journal.pone.0250918)
Supplement: S1 File — (PDF) [file pone.0250918.s001.pdf]

PLOS ONE

RESEARCH ARTICLE

No interaction between fundamental-frequency differences and spectral region when perceiving speech in a speech background

Sara M. K. Madsen[1,2]*, **Torsten Dau**[2], **Andrew J. Oxenham**[1]

1 Department of Psychology, University of Minnesota, Minneapolis, MN, United States of America, **2** Hearing Systems Section, Department of Health Technology, Technical University of Denmark, Lyngby, Denmark

* saramiay@gmail.com

OPEN ACCESS

Citation: Madsen SMK, Dau T, Oxenham AJ (2021) No interaction between fundamental-frequency differences and spectral region when perceiving speech in a speech background. PLoS ONE 16(4): e0249654. https://doi.org/10.1371/journal.pone.0249654

Editor: Qian-Jie Fu, University of California, Los Angeles, UNITED STATES

Received: October 13, 2020

Accepted: March 22, 2021

Published: April 7, 2021

Copyright: © 2021 Madsen et al. This is an open access article distributed under the terms of the Creative Commons Attribution License, which permits unrestricted use, distribution, and reproduction in any medium, provided the original author and source are credited.

Data Availability Statement: The data are available at: https://apclab.psych.umn.edu/PLOS/broadbandConditions.csv https://apclab.psych.umn.edu/PLOS/filtered.csv https://apclab.psych.umn.edu/PLOS/ReferenceConditions.csv https://apclab.psych.umn.edu/PLOS/F0DL.csv.

Funding: SM is funded by the William Demant Foundation (http://williamdemantfoundation.com/). AJO is funded by NIH grant R01 DC005216 The funders had no role in study design, data collection

Abstract

Differences in fundamental frequency (F0) or pitch between competing voices facilitate our ability to segregate a target voice from interferers, thereby enhancing speech intelligibility. Although lower-numbered harmonics elicit a stronger and more accurate pitch sensation than higher-numbered harmonics, it is unclear whether the stronger pitch leads to an increased benefit of pitch differences when segregating competing talkers. To answer this question, sentence recognition was tested in young normal-hearing listeners in the presence of a single competing talker. The stimuli were presented in a broadband condition or were highpass or lowpass filtered to manipulate the pitch accuracy of the voicing, while maintaining roughly equal speech intelligibility in the highpass and lowpass regions. Performance was measured with average F0 differences (ΔF0) between the target and single-talker masker of 0, 2, and 4 semitones. Pitch discrimination abilities were also measured to confirm that the lowpass-filtered stimuli elicited greater pitch accuracy than the highpass-filtered stimuli. No interaction was found between filter type and ΔF0 in the sentence recognition task, suggesting little or no effect of harmonic rank or pitch accuracy on the ability to use F0 to segregate natural voices, even when the average ΔF0 is relatively small. The results suggest that listeners are able to obtain some benefit of pitch differences between competing voices, even when pitch salience and accuracy is low.

The accuracy with which we are able to discriminate the pitch of a harmonic complex tone depends on the F0 and the harmonic numbers present. For F0s in the average range of speech (100–200 Hz), pitch discrimination is best (implying accurate F0 coding) when harmonics below about the 10th are present [6–10]. When these lower-numbered harmonics are present, pitch discrimination is also independent of the phase relationships between the harmonics, suggesting that these harmonics are spectrally resolved to some extent. In contrast, when only harmonics above the 10th are present in this range of F0s, pitch discrimination is poorer and is affected by the phase relationships between harmonics, suggesting that interactions occur between these spectrally unresolved harmonics [6–10].

Psychoacoustic studies of sound segregation have often been carried out with interleaved sequences of tones. Some of these studies have investigated segregation based on

and analysis, decision to publish, or preparation of the manuscript.

Competing interests: The authors have declared that no competing interests exist.

differences in pitch accuracy and have varied the accuracy by systematically varying whether resolved or only unresolved harmonics are present. Previous studies have found that stream segregation can occur with alternating sequences of tones, even if the tones consist only of unresolved harmonics [11–14]. However, the question of whether streaming is greater with resolved than unresolved harmonics has received mixed answers. In cases where the listeners' task was to segregate the streams, some studies have shown little difference in streaming between conditions containing resolved or only unresolved harmonics [11, 15], whereas another study using a similar approach found significantly greater stream segregation when resolved harmonics were present than when only unresolved harmonics were present [12]. However, in situations where the task was either neutral or encouraged listeners to integrate the sequences into a single stream, the results have been consistent across studies in showing greater segregation for complex tones containing resolved harmonics than for tones containing only unresolved harmonics [13, 14]. These findings support the idea that pitch accuracy can affect our ability to segregate sounds.

Less is known about the role of low-numbered harmonics in the context of segregating competing speech. Bird and Darwin [2] showed that lower harmonics dominate performance in a speech-segregation task based on F0 differences, but they did not test any conditions containing only high-numbered harmonics. Oxenham and Simonson [16] explored the effect of harmonic rank on speech intelligibility by comparing conditions where the target and single-talker masker had been lowpass (LP) or highpass (HP) filtered to either retain (LP-filtered) or remove (HP-filtered) the spectrally resolved components from the target and masker [16]. The LP and HP cutoff frequencies were selected to produce roughly equal performance in noise for both conditions. Surprisingly, performance in the LP and HP conditions improved by similar amounts when the noise masker was replaced by a single-talker masker with a different average F0, suggesting no clear benefit of having resolved harmonic components in the speech. However, that study only used relatively large values of average ΔF0 that according to recent F0 estimates were approximately 4 and 8 semitones (ST). Moreover, this study did not parametrically vary the ΔF0 between the target and masker. It may be that pitch accuracy is only relevant for more challenging conditions, i.e. for conditions with smaller average values of ΔF0. Thus, it remains unclear whether the effect of ΔF0 on performance is affected by the presence or absence of low-numbered, spectrally resolved harmonics. The aim of the present study was to determine whether there is an effect of spectral region, and hence pitch coding accuracy, on the ability of listeners to use average F0 differences between a target and an interfering talker to understand natural speech.

Introduction

Pitch differences between competing voices facilitate our ability to segregate speech from a background of other speakers [1–4]. It is, for example, easier to understand a female speaker in the presence of a male speaker than in the presence of another female speaker [5]. Therefore, it seems plausible that the ability to make use of pitch differences would improve with increasing fundamental frequency (F0) coding (or pitch) accuracy. Sounds whose pitch is perceived less accurately might be perceived as more similar to each other and might therefore be more likely to fuse and harder to segregate. However, the importance of pitch accuracy for understanding

speech in a speech background has not been clearly demonstrated in previous studies. The aim of the current study was to test this predicted relationship between pitch accuracy and speech segregation ability.

Methods

General methods

The experiments were conducted in a double-walled acoustically shielded booth. The stimuli were generated in MATLAB (The Mathworks, Natick, MA, USA) and were presented at a sampling rate of 48 kHz via a Fireface UCX sound card (RME, Haimhausen, Germany) and HD650 headphones (Sennheiser, Wedemark, Germany). The experimental protocols were approved by the Scientific Ethical Committees of the Capital Region of Denmark (H-16036391). All participants provided written informed consent before the experiment.

Speech experiment

Eighteen native speakers of Danish (9 female, 9 male) between 20 and 28 years of age (mean = 23.7, SD = 2.4) were tested. All participants had audiometric thresholds at octave frequencies between 250 and 8000 Hz no greater than 20 dB hearing level (HL). Speech intelligibility was tested for target sentences in the presence of a single-talker masker under HP, LP, and unfiltered (broadband) conditions. The target in each trial was a sentence from the conversational language understanding evaluation (CLUE) speech corpus [17]. The masker was created from the concatenated speech of one talker from recordings of conversations between two talkers [18], after all gaps exceeding 100 ms, non-Danish words, loud exclamations, and other sounds such as laughter were removed. The remaining 222.3 s of masker was then divided into 180 overlapping segments of 2.47 s each. The maskers for the training (30 segments) were taken from a different talker from another recorded conversation. The maskers started 500 ms before the target, ended at least 100 ms after the target, and were gated with 50-ms raised-cosine onset and offset ramps. One CLUE sentence (always the same) was presented in quiet immediately before each trial as a guide to help listeners focus on the target voice. All talkers were male. The original long-term average F0 was approximately 110 Hz for the target, 139 Hz for the masker used for testing, and 148 Hz for the masker used for training. The F0s of the maskers were manipulated in Praat [19] to obtain differences between the long-term average F0 of the target and masker (ΔF0) of 1, 3, and 5 ST for the training and 0, 2, and 4 ST for the main test. Different ΔF0s were used for training and testing to familiarize the participants with the experimental design without training the specific conditions tested. The range of tested average ΔF0s was relatively small because it was hypothesized that accurate pitch coding would be most important at small values of ΔF0. Furthermore, larger ΔF0s have been previously shown to produce similar results for both LP- and HP-filtered speech [16]. The average long-term F0 of the masker was always the same as or higher than that of the target. The speech maskers were filtered to have the same long-term spectrum as the CLUE sentences. The inherent F0 variations of both the masker and target were maintained to provide a more natural percept [20].

For the LP and HP conditions, the target, masker, and guide sentence were lowpass or highpass filtered with a 4th-order Butterworth filter after being combined. The conditions with ΔF0 of 4 ST were used as a reference, since it is the condition that is closest to the ones tested by Oxenham and Simonson [16]. Cutoff frequencies of 800 and 1500 Hz were chosen for the LP and HP conditions, respectively, based on pilot experiments that indicated they would yield similar performance. A target-to-masker ratio (TMR) of 0 dB was used for the LP and HP conditions and a TMR of -15 dB was used for the broadband conditions to obtain similar

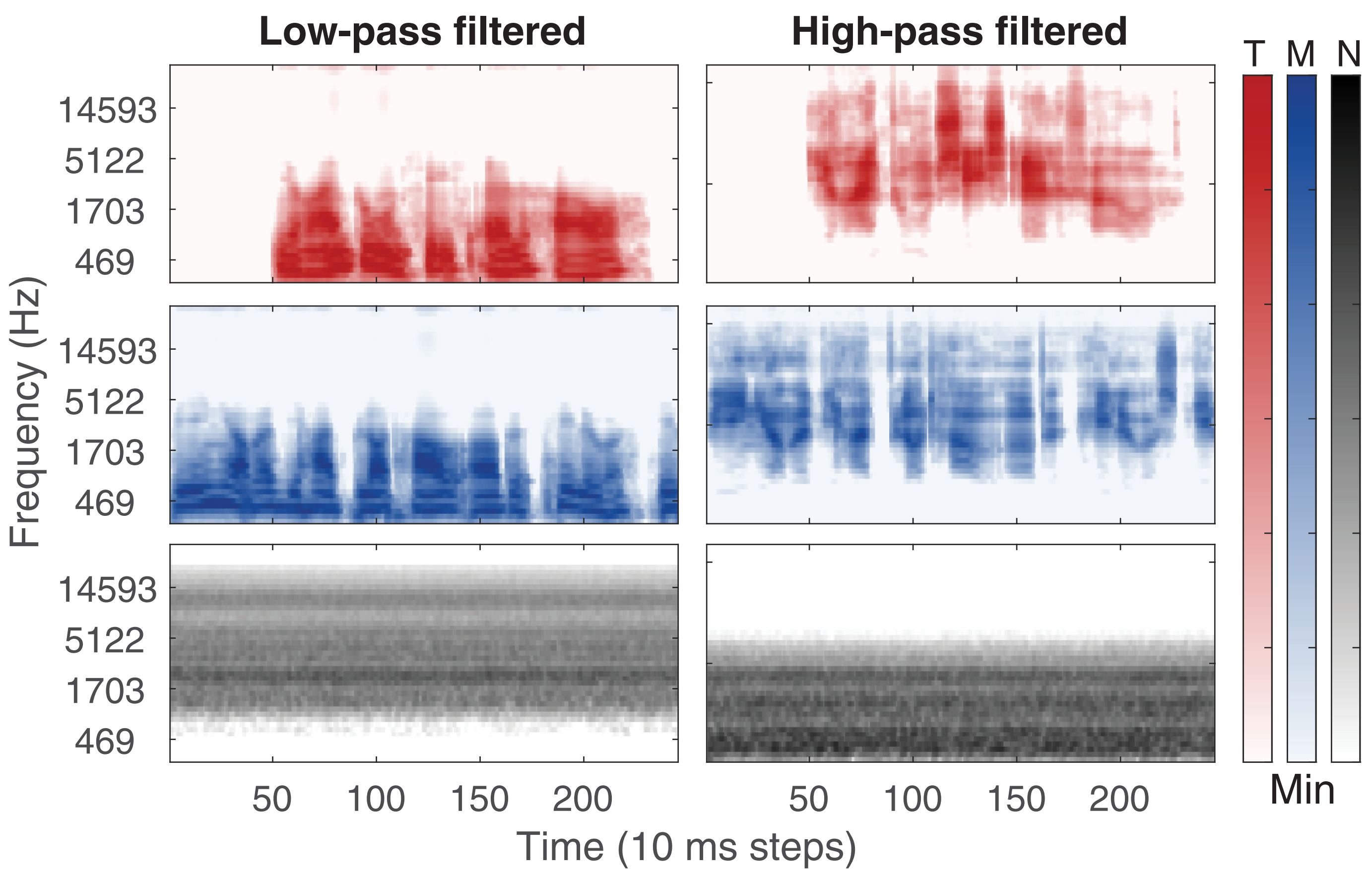

Fig 1. Lowpass and highpass filtered stimuli. Cochleagrams generated using the gammatonegram function [21] for an example of the stimuli with ΔF0 of 4 STs for the lowpass- and highpass-filtered conditions in the left and right columns, respectively. The top row shows the target (T), the middle row shows the speech masker (M) and the bottom row shows the off-band noise masker (N).

https://doi.org/10.1371/journal.pone.0249654.g001

performance for ΔF0 = 4 ST. Examples of the stimuli from the HP and LP conditions are shown in Fig 1.

A Gaussian noise, spectrally shaped to have the same long-term spectrum as the CLUE sentences (before filtering), was filtered with a 4th-order Butterworth filter and added to the filtered speech stimuli. For the LP condition, the noise was highpass filtered with a cutoff frequency of 800 Hz and for the HP condition, the noise was lowpass filtered with a cutoff frequency of 1500 Hz. The root mean squared (RMS) level of the noise before filtering was 12 dB lower than the unfiltered target speech. The target and maskers combined were presented at 70 dB sound pressure level (SPL). The purpose of this off-band noise was to limit the use of speech information in the slopes of the filters and to mask distortion products that might occur in the HP-filtered condition.

In the main test, each of the nine conditions (three filter conditions by three ΔF0s) was tested with two lists, each containing 10 sentences. The presentation order of the conditions was randomized within each of two consecutive blocks, each containing all of the nine conditions. The training consisted of three runs presented in the following order: 1) Broadband with a ΔF0 of 5 ST, presented at a TMR of -12 dB; 2) HP with a ΔF0 of 3 ST, presented at a TMR of 3 dB; 3) LP with a ΔF0 of 1 ST, presented at a TMR of 0 dB. The participants were instructed to

listen for the voice of the guide sentence and were asked to type exactly what they heard that voice say, after each trial. No feedback was provided.

F0 discrimination

The purpose of the F0 discrimination experiment was to confirm that F0 discrimination is poorer, and more dependent on component phase (indicating spectrally unresolved harmonics), for the HP stimuli than the LP stimuli. Six participants (5 female and 1 male, aged between 21 and 26 years) were tested, all of whom had also participated in the speech experiment. The F0 difference limens (F0DLs) were measured with a two-interval, two-alternative forced-choice method, where each interval contained four tones of 200-ms duration, including 20-ms ramps, presented contiguously, as in earlier studies [3, 20]. In the reference interval, all tones had the same F0, selected at random in each interval from a uniform 2-ST range centered on 131 Hz (corresponding to one standard deviation above the long-term average F0 of the target speech). In the target interval, the reference F0 was again selected at random from the same 2-ST range, with the actual F0 of the first and third tone being higher and the F0 of the second and fourth tone being lower than the reference F0. The difference in F0 between the high and low tones, ΔF0, was varied adaptively, while the F0s of the tones remained geometrically centered on the reference F0. The two intervals were separated by a 400-ms gap. Participants were asked to indicate which interval contained the variations in pitch. Feedback was provided after each trial. Each tone contained all harmonics up to 10 kHz and was filtered to have the same spectral envelope as the long-term spectrum of the target sentences. The harmonic components were added in either sine or random phase. For the random-phase conditions, the phase was chosen randomly and independently for each component in each tone from a uniform distribution from 0 to 2π.

As in the speech experiment, the tones were either lowpass or highpass filtered with a fourth-order Butterworth filter using cutoff frequencies of 800 and 1500 Hz, respectively. Moreover, as in the speech experiment, the same HP speech-shaped noise with a cutoff frequency of 800 Hz was added in the LP condition and the LP speech-shaped noise with a cutoff frequency of 1500 Hz was added in the HP condition, again to limit the audibility of distortion products. The average overall level of the tones was 70 dB SPL but the level was roved independently for each complex tone over a uniform range of 6 dB. The noise was presented at a level 12 dB below the nominal level of the tones before filtering.

Thresholds were estimated using a 3-down 1-up adaptive tracking procedure. The initial value of ΔF0 was 19.95% and it was initially decreased or increased by a factor of 2. After the second upper reversal the ΔF0 was changed by a factor of 1.26; after two more reversals, the ΔF0 was changed by a factor or 1.12 for the final six reversals. For each run, the threshold was calculated as the geometric mean of ΔF0 across the last six reversals. The experiment contained three blocks, with one run for each condition, and the order of the conditions was randomized within a block. The first run was used for training and the final thresholds were defined as the geometric mean of the two last runs.

Results

Speech perception

Speech intelligibility was defined as the proportion of words reported correctly in each condition. All deviations except obvious misspellings or homophones were considered incorrect. Additional words and differences in word order were not penalized. Results from the broadband condition are shown in the left panel of Fig 2, and results from the LP and HP conditions are shown in the right panel. Considering first only the results from the 4-ST separations in

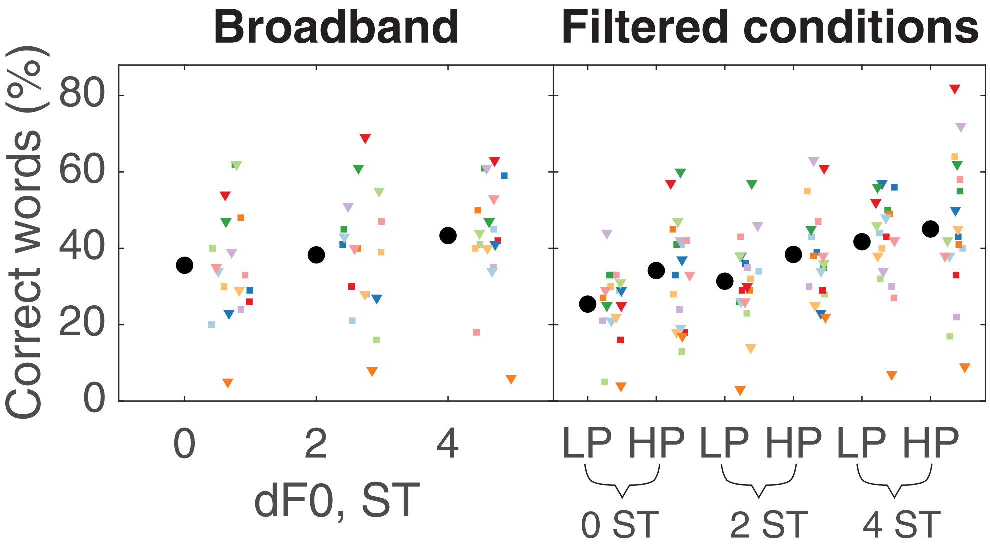

Fig 2. Results for the speech experiment. Speech scores for the broadband condition (left panel), and the lowpass- and highpass-filtered conditions (right panel). Larger black circles represent the mean across participants and the smaller colored symbols show the individual scores.

https://doi.org/10.1371/journal.pone.0249654.g002

each condition (the reference conditions), it can be seen that the mean scores were very similar. Indeed, a repeated-measures ANOVA using just the data from the 4-ST separations in the broadband, LP, and HP conditions showed no significant effect of filter condition [F(2,34) = 0.47, p = 0.63, η^2_G = 0.0081], where η^2_G is the generalized eta squared [22]. This finding confirmed that the cutoff frequencies chosen for the HP and LP conditions and the TMRs chosen for the filtered and broadband conditions yielded similar performance in the three reference conditions. The speech scores from the broadband conditions were analyzed separately, since the filtered conditions were measured at a higher TMR than the broadband conditions. For the broadband condition, there was a tendency for the scores to improve with increasing ΔF0. Analysis of the speech scores with ΔF0 as the within-subject factor showed a small but significant effect of ΔF0 [F(2,34) = 3.45, p = 0.043, η^2_G = 0.046]. Moreover, Bonferroni-corrected post-hoc tests (α = 0.0167) showed a significant difference between the conditions with ΔF0 of 0 and 4 semitones [t(34) = -2.59, p = 0.014, Cohen's d = 0.61] but not between either of the other two pairs of conditions. The right panel of Fig 2 shows individual and mean speech scores for the LP and HP conditions for ΔF0s of 0, 2, and 4 semitones. As expected, scores also generally improved with increasing ΔF0. Furthermore, the scores were generally higher for the HP than for the corresponding LP conditions, especially for ΔF0s of 0 and 2 ST. Analysis with ΔF0 and filter condition as within-subject factors showed a significant effect of both ΔF0 [F(2, 34) = 25.68, p $<$ 0.0001, η^2_G = 0.16] and filter condition [F(1,17) = 8.67, p = 0.0091, η^2_G = 0.056] but no interaction [F(2, 34) = 1.01, p = 0.38, η^2_G = 0.0074], indicating no significant difference between the low- and high-numbered harmonics in terms of their contribution to the effects of ΔF0 between competing voices.

F0 discrimination

Mean and individual F0DLs are shown in Fig 3. As expected, F0DLs were higher in the HP conditions than in the LP conditions, and phase affected F0DLs in the HP, but not in the LP, conditions. Results from a repeated-measures ANOVA on the log-transformed F0DLs

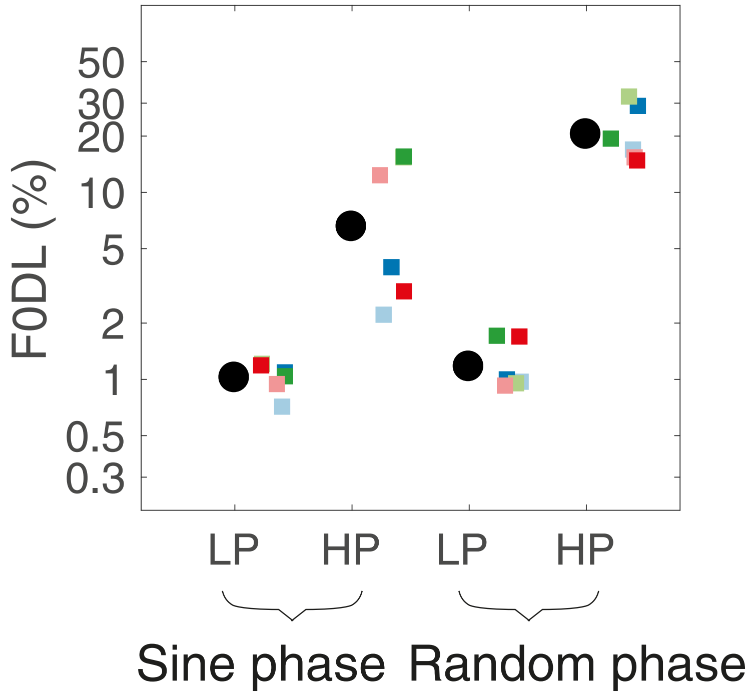

Fig 3. F0 discrimination thresholds. Thresholds for complex tones with components added in sine (left) or random (right) phase and under lowpass- or highpass-filtered conditions. Larger black circles represent mean thresholds, and the smaller colored symbols represent individual thresholds.

https://doi.org/10.1371/journal.pone.0249654.g003

confirmed significant main effects of both filter type [F(1, 5) = 117.99, p = 0.00011, η_G^2 = 0.87] and phase [F(1,5) = 11.99, p = 0.018, η_G^2 = 0.32] and a significant interaction between phase and filter condition [F(1,5) = 7.6, p = 0.040, η_G^2 = 0.23]. Bonferroni-corrected paired comparisons (α = 0.025) confirmed that the F0DL was significantly poorer for the random-phase than the sine-phase condition after HP filtering [t(10) = -4.4, p = 0.0061] but not after LP filtering [t (10) = -0.526, p = 0.95]. The results are consistent with expectations based on only high-numbered, spectrally unresolved, harmonics being present when the stimuli were highpass filtered with a cutoff frequency of 1500 Hz, as in the speech experiment. The results confirm that the F0 of the speech should have been less accurately represented under HP conditions than under LP conditions, with F0DLs higher by an order of magnitude.

Discussion

In the speech experiment, the improvement in speech scores with increasing ΔF0 is consistent with results from earlier studies [1–3, 23]. The lack of a main effect of filter type when considering the reference conditions confirms that we were successful at selecting filter cutoff frequencies that produced roughly equal performance in the LP and HP conditions when ΔF0 = 4 ST. Due to this planned equalization of overall performance, it is more relevant to compare the interaction between the filtering condition and ΔF0, or the relative slopes of the functions, rather than the absolute scores. There is a slightly steeper slope for the LP than the HP condition with increasing ΔF0, consistent with a stronger effect of ΔF0 in conditions with the more accurate pitch. However, this difference in slopes did not reach significance, as indicated by the lack of an interaction between filter type and ΔF0. The number of participants was reasonably large (N = 18) and estimated effect size of the interaction was so small (η_G^2 =

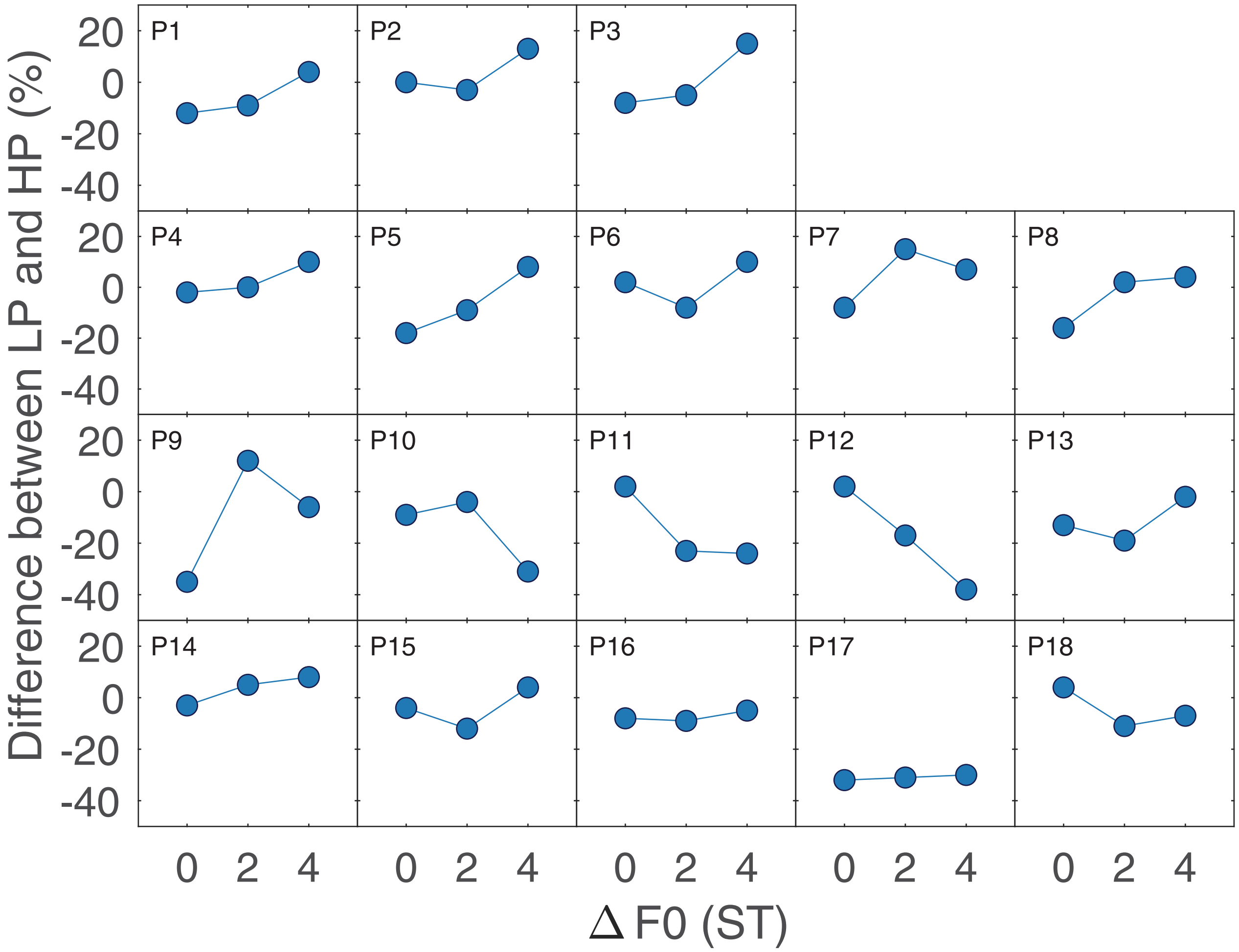

Fig 4. Difference between the lowpass-filtered and highpass-filtered conditions for each participant. A positive slope indicates a greater effect of ΔF0 for the LP-filtered than for the HP-filtered condition, as would be expected if greater pitch accuracy improved listeners' ability to take advantage of average F0 differences between the target and masker.

https://doi.org/10.1371/journal.pone.0249654.g004

0.0074) that the lack of an effect does not seem likely to be due to a lack of statistical power. To determine whether there were substantial individual differences in the influence of LP or HP filtering on the effect of ΔF0, the difference between the scores obtained in the LP and HP conditions are plotted for each participant in separate panels in Fig 4. Here, a positive slope indicates a larger effect of the F0 difference in the LP than in the HP conditions. About half of the participants (Fig 4; P1-P9) seemed to exhibit a greater benefit of ΔF0 for the LP than for the HP conditions (positive slope), but the difference was generally small and not always monotonic. Furthermore, there was considerably larger effect of ΔF0 for the HP-filtered than for the LP-filtered conditions (negative slope) for three participants (Fig 4; P10-P12) and little difference between the effect of ΔF0 for the two filter conditions for six participants (Fig 4; P13-P18). This suggests that even if there is an interaction between filter condition and ΔF0 for some participants, it is not a large or robust effect.

The lack of interaction is consistent with the results from Oxenham and Simonson [16], who found similar performance for HP- and LP-filtered conditions for both the natural and

pitch-shifted single-talker maskers used in their study. However, the lack of effect remains somewhat puzzling, given the clear effect of filtering on F0DLs. Possible explanations include the different forms of speech information conveyed in the low and high spectral regions and different temporal properties [24–27]. For instance, increased temporal sparsity at high frequencies may result in more opportunities to "glimpse" the target in the high-frequency region, resulting in more influence of differences in F0, thereby counteracting the effect of reduced pitch accuracy at high frequencies. In addition, some studies suggest that the importance of different spectral regions for speech understanding may vary across participants; such variations may help explain the individual differences found in the present study [28, 29]. Arguing against this interpretation is the fact that normal-hearing listeners and hearing-impaired listeners seem to make similar use of F0 differences between talkers when alternating speech sounds are presented to them [30], despite the fact that hearing-impaired listeners typically show poorer pitch discrimination abilities than normal-hearing listeners [31].

Another possible explanation is that, despite testing the smallest possible long-term average ΔF0 of 0 STs, the momentary differences in ΔF0, due to the F0 fluctuations, might have been too large for differences in pitch accuracy to affect speech intelligibility. However, such fluctuations are intrinsic of natural speech and it seems unlikely that smaller ΔF0s would occur in real-life situations. Thus, the results suggest that pitch accuracy within the range found in normal speech may not be a limiting factor for understanding speech in a background of other speech in real-life situations. Nevertheless, it still remains possible that even poorer pitch perception, such as that experienced by many cochlear-implant users, may explain their reduced masking release in the presence of competing talkers [32–35].

In summary, this study tested speech intelligibility in a background of a single-talker masker and found a small effect of ΔF0 but a similar relation between performance and ΔF0 in both LP and HP conditions. This outcome suggests that the differences in pitch accuracy between low-numbered and high-numbered harmonics is not a major factor in our ability to use F0 differences between competing talkers to better understand natural speech.

Author Contributions

Conceptualization: Sara M. K. Madsen, Andrew J. Oxenham.

Formal analysis: Sara M. K. Madsen.

Funding acquisition: Sara M. K. Madsen, Torsten Dau.

Investigation: Sara M. K. Madsen.

Methodology: Sara M. K. Madsen, Andrew J. Oxenham.

Project administration: Sara M. K. Madsen.

Resources: Torsten Dau.

Supervision: Andrew J. Oxenham.

Visualization: Sara M. K. Madsen.

Writing – original draft: Sara M. K. Madsen.

Writing – review & editing: Sara M. K. Madsen, Torsten Dau, Andrew J. Oxenham.

References

1. Brokx JPL, Nooteboom SG. Intonation and the perceptual separation of simultaneous voices. J Phon. 1982; 10: 23–36. https://doi.org/10.1016/S0095-4470(19)30909-X

2. Bird J, Darwin CJ. Effects of a difference in fundamental frequency in separating two sentences. In: Palmer AR, Rees A, Summerfield AQ, Meddis R, editors. Psychophysical and physiological advances in hearing. London: Whurr; 1998. pp. 263–269.

3. Madsen SMK, Whiteford KL, Oxenham AJ. Musicians do not benefit from differences in fundamental frequency when listening to speech in competing speech backgrounds. Sci Rep. 2017; 7: 12624. https://doi.org/10.1038/s41598-017-12937-9 PMID: 28974705

4. Popham S, Boebinger D, Ellis DPW, Kawahara H, McDermott JH. Inharmonic speech reveals the role of harmonicity in the cocktail party problem. Nat Commun. 2018; 9: 2122. https://doi.org/10.1038/s41467-018-04551-8 PMID: 29844313

5. Brungart DS, Simpson BD, Ericson MA, Scott KR. Informational and energetic masking effects in the perception of multiple simultaneous talkers. J Acoust Soc Am. 2001; 110: 2527–2538. https://doi.org/10.1121/1.1408946 PMID: 11757942

6. Hoekstra A, Ritsma RJ. Perceptive hearing loss and frequency selectivity. In: Evans EF, Wilson JP, editors. Psychophysics and Physiology of Hearing. London, England: Academic; 1977. pp. 263–271.

7. Shackleton TM, Carlyon RP. The role of resolved and unresolved harmonics in pitch perception and frequency modulation discrimination. J Acoust Soc Am. 1994; 95: 3529–3540. https://doi.org/10.1121/1.409970 PMID: 8046144

8. Oxenham AJ, Micheyl C, Keebler MV. Can temporal fine structure represent the fundamental frequency of unresolved harmonics? J Acoust Soc Am. 2009; 125: 2189–2199. https://doi.org/10.1121/1.3089220 PMID: 19354395

9. Bernstein JGW, Oxenham AJ. The relationship between frequency selectivity and pitch discrimination: Sensorineural hearing loss. J Acoust Soc Am. 2006; 120: 3929–3945. https://doi.org/10.1121/1.2372452 PMID: 17225420

10. Mehta AH, Oxenham AJ. Effect of lowest harmonic rank on fundamental-frequency difference limens varies with fundamental frequency. J Acoust Soc Am. 2020; 147: 2314–2322. https://doi.org/10.1121/10.0001092 PMID: 32359332

11. Vliegen J, Oxenham AJ. Sequential stream segregation in the absence of spectral cues. J Acoust Soc Am. 1999; 105: 339–346. https://doi.org/10.1121/1.424503 PMID: 9921660

12. Madsen SMK, Dau T, Moore BCJ. Effect of harmonic rank on sequential sound segregation. Hear Res. 2018; 367: 161–168. https://doi.org/10.1016/j.heares.2018.06.002 PMID: 30006111

13. Vliegen J, Moore BCJ, Oxenham AJ. The role of spectral and periodicity cues in auditory stream segregation, measured using a temporal discrimination task. J Acoust Soc Am. 1999; 106: 938–945. https://doi.org/10.1121/1.427140 PMID: 10462799

14. Grimault N, Micheyl C, Carlyon RP, Arthaud P, Collet L. Influence of peripheral resolvability on the perceptual segregation of harmonic complex tones differing in fundamental frequency. J Acoust Soc Am. 2000; 108: 263–271. https://doi.org/10.1121/1.429462 PMID: 10923890

15. Ruggles DR, Tausend AN, Shamma SA, Oxenham AJ. Cortical markers of auditory stream segregation revealed for streaming based on tonotopy but not pitch. J Acoust Soc Am. 2018; 144: 2424. https://doi.org/10.1121/1.5065392 PMID: 30404514

16. Oxenham AJ, Simonson AM. Masking release for low- and high-pass-filtered speech in the presence of noise and single-talker interference. J Acoust Soc Am. 2009; 125: 457–468. https://doi.org/10.1121/1.3021299 PMID: 19173431

17. Nielsen JB, Dau T. Development of a Danish speech intelligibility test. Int J Audiol. 2009; 48: 729–741. https://doi.org/10.1080/14992020903019312 PMID: 19626512

18. Sørensen AJ, Fereczkowski M, MacDonald EN. Task dialog by native-Danish talkers in Danish and English in both quiet and noise. 2018. Available: https://zenodo.org/record/1204951#.XU7tvS2ZN3Q

19. Boersma P, Weenink D. Praat: doing phonetics by computer (Version 5.1.3.1). 2009.

20. Madsen SMK, Marschall M, Dau T, Oxenham AJ. Speech perception is similar for musicians and non-musicians across a wide range of conditions. Sci Rep. 2019;9. https://doi.org/10.1038/s41598-018-36956-2 PMID: 30626887

21. Ellis D. Gammatone-based (auditory) spectrograms. MATLAB Central File Exchange; 2020.

22. Bakeman R. Recommended effect size statistics for repeated measures designs. Behav Res Methods. 2005; 37: 379–384. https://doi.org/10.3758/bf03192707 PMID: 16405133

23. Mesiano P, Zaar J, Bramsløw L, Pontoppidan N, Dau T. Assessing the impact of fundamental frequency on speech intelligibility in competing-talker scenarios. Proc Int Symp Audit Audiol Res. 2020;7.

24. Turner CW, Kwon BJ, Tanaka C, Knapp J, Hubbartt JL, Doherty KA. Frequency-weighting functions for broadband speech as estimated by a correlational method. J Acoust Soc Am. 1998; 104: 1580–1585. https://doi.org/10.1121/1.424370 PMID: 9745741

25. Calandruccio L, Doherty KA. Spectral weighting strategies for sentences measured by a correlational method. J Acoust Soc Am. 2007; 121: 3827–3836. https://doi.org/10.1121/1.2722211 PMID: 17552730

26. Gilbert G, Micheyl C. Influence of competing multi-talker babble on frequency-importance functions for speech measured using a correlational approach. Acta Acust United with Acust. 2005; 91: 145–154.

27. Apoux F, Bacon SP. Selectivity of modulation interference for consonant identification in normal-hearing listeners. J Acoust Soc Am. 2008; 123: 1665–1672. https://doi.org/10.1121/1.2828067 PMID: 18345854

28. Doherty KA, Turner CW. Use of a correlational method to estimate a listener's weighting function for speech. J Acoust Soc Am. 1996; 100: 3769–3773. https://doi.org/10.1121/1.417336 PMID: 8969478

29. Lutfi RA, Doherty KA, Oh E. Psychometric functions for the discrimination of spectral variance. J Acoust Soc Am. 1996; 100: 2258–2265. https://doi.org/10.1121/1.417935 PMID: 8865634

30. David M, Tausend AN, Strelcyk O, Oxenham AJ. Effect of age and hearing loss on auditory stream segregation of speech sounds. Hear Res. 2018; 364: 118–128. https://doi.org/10.1016/j.heares.2018.03.017 PMID: 29602593

31. Bernstein JG. Pitch perception and harmonic resolvability in normal-hearing and hearing-impaired listeners. MIT, Cambridge, MA. 2006.

32. Fu QJ, Nogaki G. Noise susceptibility of cochlear implant users: the role of spectral resolution and smearing. J Assoc Res Otolaryngol. 2005; 6: 19–27. https://doi.org/10.1007/s10162-004-5024-3 PMID: 15735937

33. Croghan NBH, Smith ZM. Speech Understanding With Various Maskers in Cochlear-Implant and Simulated Cochlear-Implant Hearing: Effects of Spectral Resolution and Implications for Masking Release. Trends Hear. 2018;22. https://doi.org/10.1177/2331216518787276 PMID: 30022730

34. Nelson PB, Jin SH, Carney AE, Nelson DA. Understanding speech in modulated interference: cochlear implant users and normal-hearing listeners. J Acoust Soc Am. 2003; 113: 961–968. https://doi.org/10.1121/1.1531983 PMID: 12597189

35. Stickney GS, Zeng FG, Litovsky R, Assmann P. Cochlear implant speech recognition with speech maskers. J Acoust Soc Am. 2004; 116: 1081–1091. https://doi.org/10.1121/1.1772399 PMID: 15376674
